# Real-Time Lane Region Detection Using a Combination of Geometrical and Image Features

**DOI:** 10.3390/s16111935

**Published:** 2016-11-17

**Authors:** Danilo Cáceres Hernández, Laksono Kurnianggoro, Alexander Filonenko, Kang Hyun Jo

**Affiliations:** Intelligent Systems Laboratory, Graduate School of Electrical Engineering, University of Ulsan, Ulsan 44610, Korea; danilo@islab.ulsan.ac.kr (D.C.H.); laksono@islab.ulsan.ac.kr (L.K.); alexander@islab.ulsan.ac.kr (A.F.)

**Keywords:** real-time lane region detection, collision risk region, lane marking hybrid-based strategy, hierarchical fitting model, distance-based color-dependent clustering

## Abstract

Over the past few decades, pavement markings have played a key role in intelligent vehicle applications such as guidance, navigation, and control. However, there are still serious issues facing the problem of lane marking detection. For example, problems include excessive processing time and false detection due to similarities in color and edges between traffic signs (channeling lines, stop lines, crosswalk, arrows, etc.). This paper proposes a strategy to extract the lane marking information taking into consideration its features such as color, edge, and width, as well as the vehicle speed. Firstly, defining the region of interest is a critical task to achieve real-time performance. In this sense, the region of interest is dependent on vehicle speed. Secondly, the lane markings are detected by using a hybrid color-edge feature method along with a probabilistic method, based on distance-color dependence and a hierarchical fitting model. Thirdly, the following lane marking information is extracted: the number of lane markings to both sides of the vehicle, the respective fitting model, and the centroid information of the lane. Using these parameters, the region is computed by using a road geometric model. To evaluate the proposed method, a set of consecutive frames was used in order to validate the performance.

## 1. Introduction

The automotive industry is currently experiencing strong growth. Factors behind this are increasing congestion, environmental issues, wireless network expansion, efficiencies in automotive logistics, and demand for driver, passenger, and pedestrian safety. In that sense, advanced driver assistance systems (ADAS) have shown a very significant development since the early 1970s. ADAS focuses on vision-based applications, with examples including lane departure warnings, lane keep assist, pedestrian detection, and collision avoidance. Although recent technological advancements show significant development results, there are still problems to solve. For example, actively-researched problems include gauging uncertainty in a static/dynamic environment, improving processing time response, and managing road complexity.

Vision-based lane marking approaches focus primarily on the color-pattern changes between the road surface and pavement marking materials [1,2,3,4,5,6]. This difference in color is used to detect lane markings by extracting the features, such as edge and color information. Once the lane marking features are extracted, a model fitting is implemented taking into consideration the lane marking geometry design, specifically width and length. Last but not least, processing time plays an important role in decision making. By considering color-based methods, researchers in [1,2,3] focused on the task of lane marking detection using the HSI (hue, saturation, and intensity) color model. The algorithmic strategy proposed by the authors [1,2] shows good performance in detection and processing time. However, this algorithm extracts the lane marking using only the color information belonging to the markings, which is not accurate enough considering that there is a wide range of traffic signals on the road with the same color information (crosswalk, stop lines, one-way streets, merging/diverging traffic, etc.) without also taking into consideration the noise information (spilled paint on the road or surface with colors that belong to the lane marking). Researchers in [3] proposed a high-precision lane-level localization using a stereo camera. After the rectification between stereo images is finished, the algorithm uses the intensity value from HSI color space, and then the edge information is extracted. Unlike the previously mentioned research, these authors proposed additional detectors in order to avoid false detection (humps, crosswalks, traffic word signals, arrows). Once the edges are extracted, the authors proposed a hyperbola fitting to search the pixels belonging to the lane marking within the image. To solve vehicle localization problems, the authors use the random sample consensus (RANSAC) algorithm. Although the algorithm provides a good estimate of reliability due to the developed strategy, integration of these steps is time consuming.

Researchers in [4,5] give examples of edge-based methods. Mammeri et al. [4] proposed a lane marking localization by combining the maximally stable extremal region (MSER) and Hough transform (HT). MSER is used to extract the stable region given by lane markings, traffic signs, etc. Once the regions are extracted, the algorithm refines the previous results. Here, the task entails line detection using a probabilistic progressive Hough transform to extract the line segments with a minimum required length. Then, the line segments within 10${}^{\circ }$ and 85${}^{\circ }$ are considered lane marking candidates, and the rest of the segments are removed. The proposed method shows reliable results; however, given that they depend on the environment, the algorithm’s response is not stable (Tables 3 and 4 in [4]). Additionally, the strategy is only suitable for a straight lane marking regions with a low velocity and high camera frame rate constraints. Researchers in [5] proposed an efficient lane detection method using spatiotemporal images. After the alignment between the frames is performed, the lane points are detected using the most dominant straight line defined by the HT. Then, a weighted least-squares fitting is used as a fitting model. The algorithm has good performance with high processing time. Similarly, as gauged by authors in [1,2], the algorithm shows some error in regions such as bumps.

Researchers in [7] introduced a laser scanner application to estimate the vehicle heading angle. To do that, the authors proposed identifying the lane within the road surface by finding differences between road and marking materials. Consequently, the lane region, the lane marking, the centroid of the lane width, the road geometry as well the vehicle heading angle are iteratively estimated. Firstly, for a laser line-scanner input range data, the set of points within the region of interest is extracted. The region of interest is defined as 20 m × 4 m, the square box is located 10 m to the left and right of the laser sensor and between 10 m and 14 m in front of the laser. Secondly, in order to detect the road surface, an unsupervised density-based spatial clustering is implemented. As a result, the number of lanes within the road are detected. Thirdly, the lane markings are detected to improve the accuracy of the previous step. This is done by finding the discontinuity caused by the change between surface, road to lane marking or vice-versa. Fourthly, the centroid of the lane is the center point computed as the difference between the left and right lane marking located ahead of a vehicle (lane width). Fifthly, using the centroid information, the road geometry parameters are extracted; the deflection angle *θ*, the chord length d${}_{a}$, and the vehicle heading angle *δ* are computed. Although the algorithm shows a good performance and accuracy, it may encounter problems in regions where lane markings are considerably deteriorated.

All the research introduced above shows reliable results for use in the field of advanced driver assistance systems or autonomous navigation. However, all of these methods are still facing problems, either in processing time or performance. Consequently, in order to improve the current algorithms, a vision-based real-time lane detection algorithm is proposed for application in intelligent vehicles. In that sense, the presented method does not just take into consideration the features of color, edge, and length lane marking information, but also combines them, resulting in a speed-dependent, color-edge hybrid lane marking detection algorithm.

To summarize, the main steps of the proposed method are described below. Regions within the image are classified into categories of collision risk, zero collision risk, and regions above the horizon. The proposed algorithm focuses primarily on the collision risk region, which is extracted taking into account the vehicle speed (considered as an important key), and the region of interest (ROI) increases as the speed increases and vice versa. The color and edge lane marking features are extracted by analyzing the color and width information. Then, by combining both features, the midpoints of the lane markings are extracted. This process plays an important role, and, because of this strategy, the midpoints are extracted if they belong to lane marking types; otherwise, no action is carried out. In essence, other marking types such as crosswalks, line stops, and traffic signals on the road surface are rejected. The algorithm is not sensitive to these types of marking. This strategy reflected a substantial decrease in processing time compared with the current lane marking algorithms, in which additional processing should be performed in order to remove the noise given by different types of markers. Then, using the midpoint features, a hierarchical fitting model is implemented. A lane marking detection algorithm that solely relies on a line fitting model is not strong enough due to various road environment scenarios. On the other hand, based on the processing time as well as the road geometry design, implementation of a polynomial fitting model along the vehicle trajectory is not the best solution. Using the proposed sensor setup, it was observed that around 5% of the lane markings within the image can be defined using a curve fitting model, while a line fitting model should be used for the rest. Consequently, to solve this problem, a hierarchical line/curve fitting model has been proposed. These strategies allow the algorithm to avoid the use of expensive computation when it is not required. Finally, the lane region is estimated by using the line/curve fitting parameters.

## 2. System Overview

**Figure 1:**
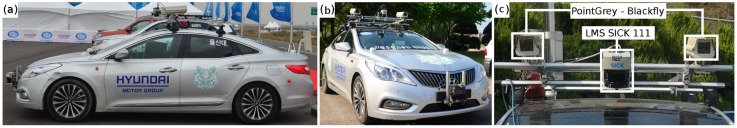
Vehicle data acquisition platform. (**a**) shows the lateral view of the vehicle; (**b**) shows the front view of the vehicle; and (**c**) shows the sensors located at the top of the vehicle at a height of approximately 1.8 m. The vehicle platform was equipped with Point Grey BlackFly cameras (Point Grey Research Inc., Richmond, BC, Canada) and a laser SICK Laser Measurement Sensor (SICK Ltd., Richmond Hill, ON, Canada).

**Figure 2:**
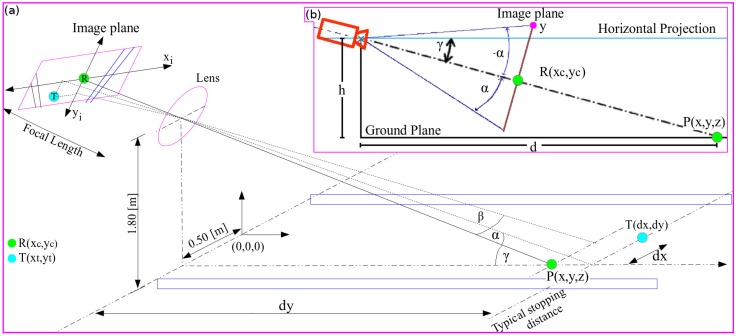
Sensor coordinate system. (**a**) shows the perspective projection; and (**b**) shows the lateral projection. The center of the coordinate system is located at the vehicle centerline, 2.3 m from the vehicle front bumper. The camera is located at a height of 1.8 m, 0.5 m to the right of the vehicle centerline. The camera is tilted down at 9${}^{\circ }$ with respect to the horizontal projection. The laser sensor is located at the vehicle centerline at the height of 1.7 m, tilted down 7.5${}^{\circ }$.

Figure 1 and Figure 2 show the sensor configuration used to gather the road imagery data, as well as the vehicle platform. The camera is located at a height of 1.8 m and tilted down at an angle of 9${}^{\circ }$ with respect to the horizontal projection, 2.3 m from the vehicle front bumper, and 0.5 m to the right of the vehicle centerline.

Assuming the vehicle undergoes planar motion, the geometry of the model is described as follows: *h* is the height of the camera above the ground, *d* is the distance between the camera at ground level and the point P(x,y,z) in the real world, $R(x_{c},y_{c})$ is the point P(x,y,z) projected to the center position of the image plane through the optical axis of the camera, T(dx,dy,dz) is the centroid of the lane at the ground level, *γ* is the angle between the optical axis of the camera and the horizontal projection, *α* is the angle formed by scanning the image from top to bottom with respect to the center point of the image $R(x_{c},y_{c})$, $y_{i}|i=1,\ldots ,256$ is the pixel position located at $x_{c}$, $\left(x_{c},y_{c}\right)$ is the pixel located at the center of the image plane, $f_{p}$ is the focal length in pixels, $f_{mm}$ is the focal length in millimeters, $I_{w}$ is the image width in pixels, and $S_{mm}$ is the CCD or CMOS image sensor width in millimeters. The vehicle data were acquired by a National Instrument PXI Express module (National Instruments Corporation, Austin, TX, USA). Additionally, a laser measurement system (LMS) was mounted on the vehicle centerline at a height of 1.7 m and tilted down at 7.5${}^{\circ }$ with respect to the horizontal projection.

Contrary to other studies [4,6], the extracted ROI is vehicle speed-dependent. To extract the ROI, the typical stopping distance is used (according to the Department for Transport and Driver and Vehicle Standards Agency of the Government of the UK). The purpose of defining the ROI using this strategy is two-fold: reducing the processing time using a dynamic speed-dependent ROI and determining the region for collision avoidance.

**Figure 3:**
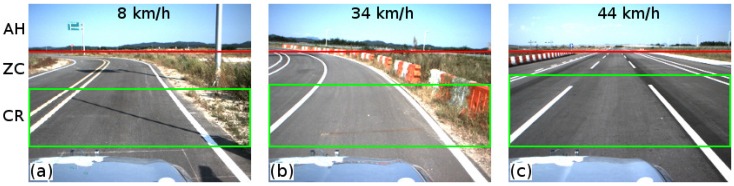
Road region of interest estimation, based on the speed of the vehicle. (**a**–**c**) show the region of the interest at various speed of the vehicle. The **red line** shows the region above the horizon (AH). The **green square** shows the region of interest, which depends on the vehicle speed. This is defined as collision risk region (CR). The region between the AH and the CR is defined as the zero collision risk region (ZC).

Figure 3 shows the road region described using the proposed sensor setup and the vehicle speed. From the sensor setup, the region over the horizon is defined (region over the red line in Figure 3). The zero collision risk region is defined as the region between the horizon and the collision risk region (between the red line and the middle green line in Figure 3). The collision risk region is extracted using the vehicle speed (green box in Figure 3). Hence, the region is defined by using a model-based typical stopping distance [8].

## 3. Proposed Method

**Figure 4:**
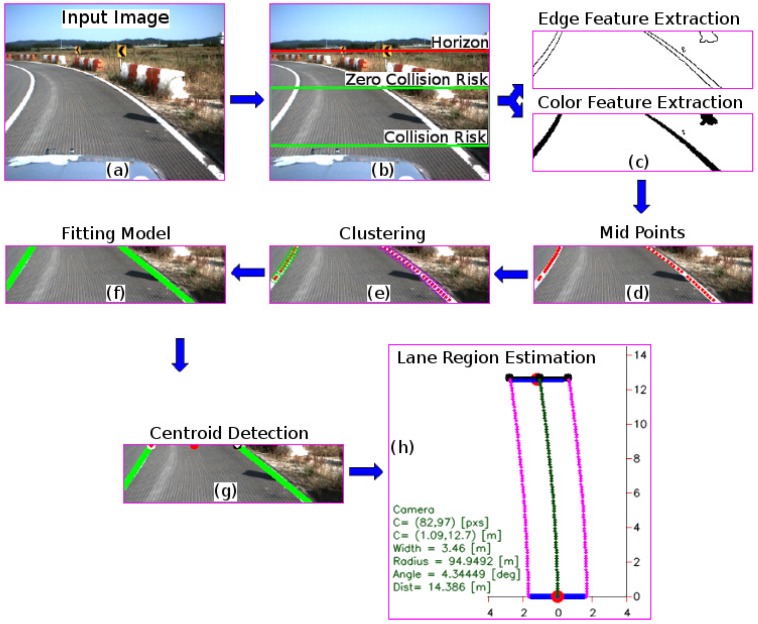
Flow diagram of the real-time lane region detection based on multi-features using a monocular camera from a frame. (**a**) shows the input color image; (**b**) shows the result of the region of interest extraction. The section of the image above the **red line** indicates the horizon region. The zero collision risk region is located between red line and the **green square**. The region within the **green square** is the collision risk region (dependent on vehicle speed), the region used in this approach; (**c**) shows the lane marking feature extraction. The **top** image shows the result of extracting the edge by using Otsu’s thresholding method. The **bottom** image shows the binary image result of extracting by using a color probability strategy; (**d**) shows the midpoints extraction method, a hybrid method which combines both the color and edge features; (**e**) shows the clustering result using a distance-based color-dependent method; (**f**) shows the fitting result. For each detected cluster, the best fitting line model is computed. Then, if the sum of the distance errors between the fitting line and the cluster is less than or equal to 15 pixels, a straight lane marking was detected; otherwise, a curve fitting model is implemented using the Lagrangian interpolation polynomial approximation; (**g**) shows the result of the centroid as well as the lane marking ending points extraction; and (**h**) shows the result of the road geometry estimation for the current frame using the centroid information.

A method to detect the lane region in real-time using a multi-feature extraction based on a vehicle speed-dependent strategy is introduced. Figure 4 shows the flow diagram of the proposed approach. The algorithm starts by extracting the ROI for a given input image using the current vehicle speed information. Then, the color and edge features are extracted, and are subsequently used in a hybrid method to extract the midpoint within the lane marking candidate. Once the midpoints are extracted, a distance-based color-dependent clustering model is applied. Hence, a fitting model is used to extract the lane marking for both lines and curves. Lastly, the centroid information, as well as the lane marking position within the image, are extracted. Finally, using the previous extracted information, the lane region is defined by computing the road geometry model. Therefore, this study aimed to detect the lane region for intelligent vehicle applications in which real-time decision making is required.

### 3.1. Lane Marking Estimation

**Figure 5:**
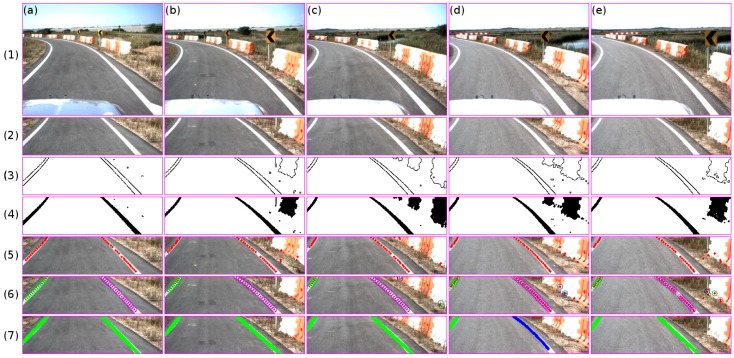
Lane marking results. (**a**–**e**) show a set of input images captured along the vehicle trajectory and their respective result of each step (from 1 to 7). The **first row** shows the input images. The **second row** shows the region of interest extraction using the speed of the vehicle. The **third and fourth rows** show the edge and color mask results. The **fifth row** shows the middle point extraction. The **sixth row** shows the clustering results. The **seventh row** shows the line/curve fitting model results.

A vision-based lane marking detection method was introduced by Cáceres et al. in [8]. The authors presented the results for the lane marking estimation using a monocular camera. Firstly, the horizon is defined off-line by using the trigonometric relationship between the camera and the target (see Figure 1 and Figure 2). Secondly, the ROI is extracted by using the vehicle typical stopping distance. It should be noted that the region which belongs to the collision risk region is used in the ROI in this approach, as shown in Figure 3 and Figure 4. The remaining regions will be used in further research. Thirdly, after the ROI has been defined, the edges are extracted using the red channel; this is because both yellow and white lane markings show a good contrast in the red channel [9,10]. This is accomplished by using Otsu’s method [11] and a statistical threshold-dependent parameter. The statistical threshold-dependent parameter helps the algorithms to deal with problems arising in the presence of shadows or other artifacts, as long as they do not appear within the image over a long period of time. Then, the edges are extracted using the Canny edge detector [12]. Fourthly, color pixels that belong to the lane marking are extracted. To do this, the probability density function (PDF) for the lane marking is created. The PDF is built for the cases of lane marking with or without presence of shadows using the red, green, and blue (RGB) color model and the saturation (S) from the hue, saturation, and value (HSV) color model. Then, the intersection is performed using the detection from each channel. Using the intersection result, the binary mask is created. As a result, for each lane marking color type (white and yellow), a mask is extracted. Finally, both masks are combined using the union between them. Fifthly, a lane marking hybrid-based strategy was implemented. The idea relies on the use of both the edge and color image results to extract the midpoint candidate. It is assumed that for each lane marking, there is a midpoint along the *x*-axis. The proposed strategy is described below. The image is scanned horizontally every *p* pixels along the *y*-axis; in the current algorithm, p is set to 3. The middle points between the consecutive edge points are extracted. Then, a Boolean AND operation is performed between the mid-point candidate and the color mask image. For all the returned true values, the pixel coordinate position is stored. Finally, to define the set of clusters, a line-based color strategy is introduced in this approach. The distance, as well as line parameters are extracted for all of the mid-point candidates. Sixthly, at this step, all of the clusters are defined. Hence, the task consists of defining the function for each given cluster for both the line and curve models. Focusing on implementing a real-time application, a hierarchical fitting model is proposed instead of using a multi-fitting model [13]. First, for each cluster, a linear model is computed. Two points are chosen randomly at each iteration (n), where *n* is computed using the total amount of points (TAP) within the cluster, computed as follows: $n=\frac{TAP}{2}$. Then, the sum of the error distances is computed for all of the line fittings. The smallest error is chosen as the best fit if and only if the error is less than 15 pixels; otherwise, the curve fitting model is computed based on an algebraic property of Lagrangian interpolation polynomial approximation. Figure 5 shows the proposed lane marking strategy results (for more details, see Cáceres et al. in [8]).

### 3.2. Lane Region Detection

**Figure 6:**
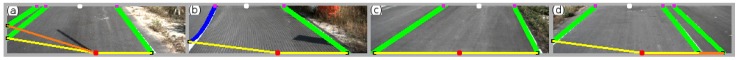
Centroid and lane marking ending points extraction. (**a**–**d**) show the result of the point extraction for a set of frames. Points in **white** show the centroid of the lane region. Points in **magenta** and **black** show the **top** and **bottom** pixel position, respectively, for each detected line/curve. Points in **red** show the center at the bottommost part of the image used to estimate the shortest distance. Lines in **yellow** show the nearest distance to the left and right side. Lines in **orange** show the largest distance to the left and right side.

At this step, the lane markings are described using line/curve fitting models. In that sense, the line/curve parameters are stored, as well as the points located at the top and bottommost part of the image. Therefore, the purpose of this step is to estimate the number of lane markings to each side of the vehicle and the centroid of the lane road. Since the fitting was already computed, the parameters for each line/curve are already known. Furthermore, in this approach the distance between the fitting model and the bottom center part of the image is computed. To this end, the nearest fit models are chosen, one to each of the left and right sides, as well as the pixel positions located at the top-most and bottom-most parts of the image. Figure 6 shows the result of this step.

**Figure 7:**
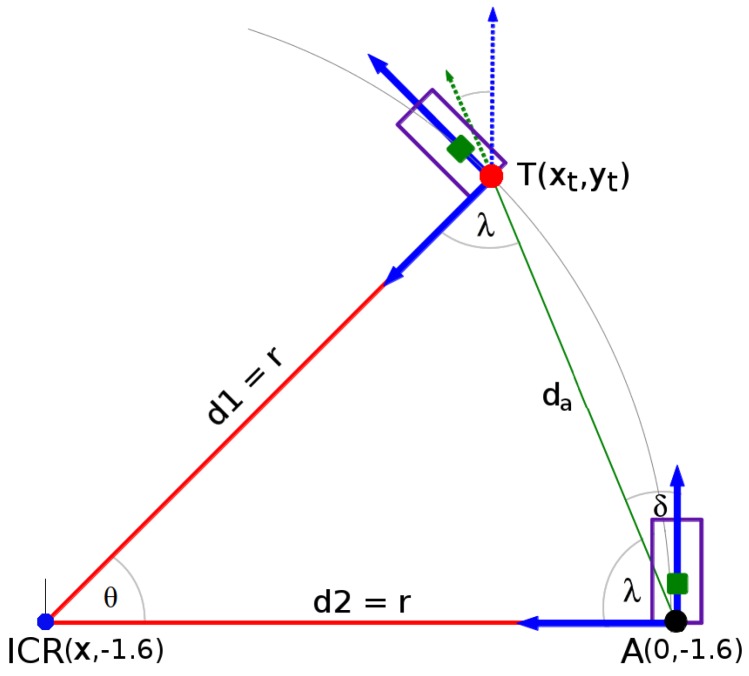
Road geometry model.

Once the centroid of the lane within the image is extracted (a pixel coordinate), the next step entails estimating the 3D coordinate at the ground level. To compute the distance using a monocular camera, several approaches have been proposed, for example by authors in [14,15,16,17]. To estimate the distance in this study, the method proposed by Fernandes et al. [17] was used, to compute the centroid. Then, the centroid position of the lane and the the lane marking points located at the topmost part and bottommost part of the lane marking were computed as follows:

(1) $$\alpha =\frac{y_{c}-y_{t}}{f_{p}};\;\beta =\frac{x_{c}-x_{t}}{f_{p}},$$

(2) $$d_{y}=\frac{h}{\tan(\gamma +\alpha )},$$

(3) $$d_{x}=\frac{d_{y}\cos\left(\alpha \right)\tan\left(\beta \right)}{\cos(\gamma +\alpha )},$$

where $f_{p}$, *α*, *β*, *γ*, $d_{y}$, $d_{x}$, $R(x_{c},y_{c})$, $T(x_{t},y_{t})$ are described in the system overview section (see Figure 2). Then, the next step consists of extracting the lane region detection following the circular motion criteria. The task consists of calculating the curvature of the circle model. To do that, the distance ahead of the vehicle as well as the heading angle formed between the centroid of the lane (previously computed) and the vehicle rear-axle should be computed. This is computed geometrically using the model shown in Figure 7, as follows:

(4) $$d_{a}=\sqrt{x_{t}^{2}+{\left(y_{t}+1.6\right)}^{2}},$$

$$\begin{array}{cc} d_{1} & =d_{2},\\ X & =-\frac{x_{t}^{2}+{\left(y_{t}+1.6\right)}^{2}}{2x_{t}},\\ r & =d_{1}=d_{2}=\left|X\right|, \end{array}$$

(5) $$\theta =2\delta \;;\;\delta =\sin^{-1}\left(\frac{d_{a}}{2r}\right),$$

where $d_{a}$ is the Euclidean distance between the point $T(x_{t},y_{t})$ and the rear-axle position located at A(0,−1.6), and $d_{1}$ and $d_{2}$ are the distances between the center of the circle model (called the instantaneous center of rotation (ICR)) and the target point $T(x_{t},y_{t})$ and the rear-axle point A(0,−1.6), respectively; here, both distances refer to the radius *r*, while *θ* is the deflection angle (which refers to the road geometry), and *δ* is the vehicle heading angle.

## 4. Experiments

The program was built in C++ and compiled in Ubuntu 12.04 using an Intel Core i7-4770 CPU, 3.40 GHz, with 8 GB of RAM. A total of 3781 frames with a total of 8617 lane markings were used, with single, double and broken lane markings on both straight and road scenes. The image size used in this algorithm was 256 × 204. The camera sensor was mounted at the top of the vehicle at 1.8 m and tilted down 9${}^{\circ }$. The vehicle moves in a speed range of [5–45] km/h, acquired by a National Instrument PXI Express module. The algorithm was tested using images gathered at the Korea Automobile Testing & Research Institute (KATRI), and they contain sand, bushes, and barriers, as well as spilled white paint along the lane markings. To test the algorithm, statistical analysis was performed to verify the reliability of the proposed method.

### 4.1. Lane Marking Evaluation

**Figure 8:**
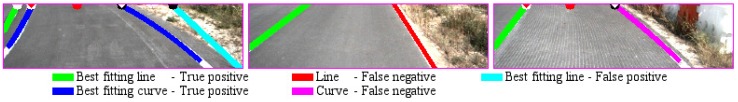
False positive and negative results. The left image shows a false positive case where the rightmost lane marking in **cyan** was detected due to the fact that the midpoints from the sand region were extracted as candidates in Section 3.1 steps three and four (see Figure 5 and Figure 9 rows 3, 4 and 5). The last two images show the case of a false negative; in this case, tracking using the previous line/curve fit model is implemented. The line in **red** shows the results of the tracking, while the **magenta** shows the results for the curve fitting. In the middle image, the false negative result was due to the fact that the lane marking edges were outside the range during the midpoint extraction process. This happened because there was spilled paint along the lane marking. In the case of the right image, the algorithm was not able to detect the lane marking because of the deterioration of the markers.

This task consists in evaluating the lane marking result of the proposed algorithm with respect to the lane marking ground truth (manually labeled). In essence, the evaluation describes the feasibility of the proposed lane marking detection strategy—in other words, how effectively the lane marking algorithm is able to detect correctly or remove lane marking candidates from the region of interest using the proposed hybrid color-edge feature method along the distance-color dependence probabilistic method and the hierarchical fitting model explained in Section 3.1 (see Figure 8). The road surface data, tested in this approach, has the following characteristics: double lane marking to the left and single lane marking to the right, single lane marking to both sides, single lane marking to the left and broken double lane marking on the right side, single lane marking to the left and channelizing lane markings to the right.

To evaluate the lane marking results, sensitivity (St), specificity (Sp), accuracy (A), and the false positive (FPs) and negative frames per second (FNs) rate metrics were used. The sensitivity measures the correct detection of the candidate as a lane marking, while the specificity measures the correct rejection of the false candidate as a non-lane marking. The accuracy describes how well the presented method correctly classifies the lane marking. FPs and FNs indicate the amount of false positives or negatives that occur in a second. They are computed as follows:

(6) $$St=\frac{TP}{TP+FN}\;;\;Sp=\frac{TN}{TN+FP}\;;\;A=\frac{TP+TN}{TP+FP+FN+TN},$$

(7) $$FPs=\frac{FP*PT}{TF}\;;\;FNs=\frac{FN*PT}{TF},$$

where TP (true positive) indicates that candidates are correctly detected, TN (true negative) indicates that candidates are correctly rejected, FN (false negative) indicates that lane markings were incorrectly rejected, and FP (false positive) indicates that lane markings were incorrectly identified, TF is the total amount of frames, PT is the processing time.

Table 1 shows the result of the proposed algorithm tested in roads with single and double lane markings, combining all of them. Although the algorithm was tested in a road with the presence of shadows, luminance problems, grass, white sand, and deterioration of pavement markings, as well as without the presence of shoulders and curbs, the results show good performance.

To overcome the problem given by scenes that cause false positives and false negatives, a tracking method was implemented using the line/curve model for each lane marking. The idea relies on the fact that lane markings cannot abruptly change their line/curve parameters between two consecutive frames (under the real-time assumption). Furthermore, in the case of no detection, the previous line/curve model is used to scan the area. Hence, the algorithm searches for color information that belongs to the lane marking. The tracking stops once the line/curve parameters are detected in the new frame, or after more than 25 frames have passed (based on observation). For future work, we will stop the process of tracking based on the current vehicle speed. Figure 8 shows the results of the proposed tracking implementation. With respect to the problems given by the change of illumination or shadows, it is noted that this condition was taken into consideration during the PDF training data. However, in the presence of strong shadows and/or changes in the illumination, the current algorithm will encounter problems in detection (a common problem in vision-based applications). In our previous study [18,19], the pavement markers were used as features for solving the problems of lane marking and crosswalk detection using a laser. Furthermore, to overcome the problem of shadows and illumination, a fusion sensor will be implemented.

**Figure 9:**
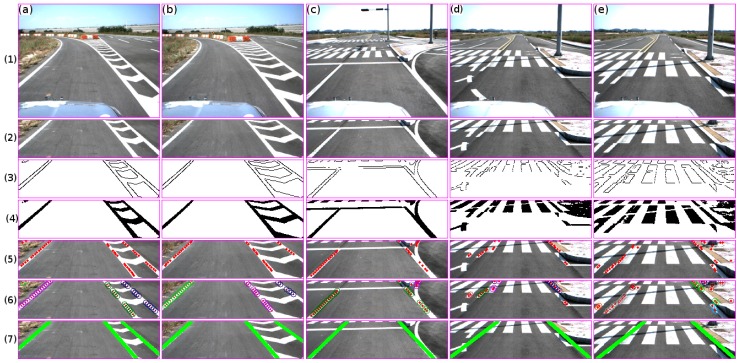
Lane marking results in regions that contain different markers such as **white** channelizing lines (**a**,**b**), stop lines (**c**), and crosswalk pavement markers (**d**,**e**). The rows show the results at each step. The first row shows the input image. The second row shows the road region of interest. The third and fourth rows show the binary image for both the edge and color probability method. The fifth row shows the extracted set of midpoints for each frame. The sixth row shows the result of the clustering process. The seventh row shows the line/curve fitting result.

Similarly, the algorithm was tested on road regions that contain different markers such as white channelizing lines, stop lines, and crosswalk pavement markers. These types of markers usually make lane marking vision-based applications fail [4,13,20]. The good performance is due to the midpoint extraction points, in which points are extracted if the distance between two consecutive edges along the *x*-axis are within the lane marking range in pixels. Basically, due to the sensor configuration, the midpoints are chosen as candidates if they are within the range of 4–12 pixels (see Figure 9).

In all of these cases, the midpoints refer to the lane marking candidate. The results in Figure 9 can be summarized as follows:

- Stopping lane markings: this type of marker does not affect the performance, since the distance between the starting and ending of the edge point is larger than the distance constraints.
- Channelizing lane markings: the midpoints are extracted from the region that belongs to the distance constraints. Using the set of extracted points and the clustering strategy, the lane markings are extracted; see columns 1 and 2 in Figure 9. In the first two columns, three clusters were detected, each one belonging to each lane marking.
- Crosswalk markings: similarly to the stopping lane marker, these markers do not affect the algorithm due to the distance constraints. Columns 4 and 5 in Figure 9 show the performance. It should be noted that the lane markings were detected due to the presence of broken markings before the crosswalk region as well as the reflection of the sun within the curb region; see the input image in Figure 9 row 1.

Finally, Table 2 shows the error fitting in both line/curve fitting results. In the proposed method, a line/curve is detected if the error is less than or equal to 15 pixels.

### 4.2. Lane Region Evaluation

As mentioned in Section 3.2, the lane region is estimated by using the vehicle typical stopping distance and the road geometry model. This step involves evaluating the required parameters to describe the lane within the collision risk region (see Figure 3), which is an important task in intelligent vehicle applications to improve driver/pedestrian safety (e.g., obstacle-avoidance, guidance navigation, lane keeping assist systems, etc.).

#### 4.2.1. Centroid of the Lane

**Figure 10:**
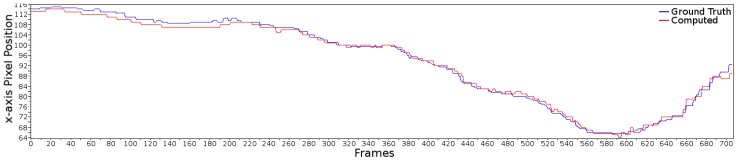
Centroid evaluation. The figure shows the comparison in pixels between the ground truth centroid (labeled by hand) and the estimated one. The ground truth is shown in **blue** while the estimated is shown in **red**.

The first parameter to evaluate is the centroid estimation of the lane region within the image. The centroid position was iteratively extracted by taking the inner edge of the lane marking, located to the left and right sides of the lane width (in pixels) for each frame. In the case of the ground truth (manually labeled), the middle point of the lane width along the *x*-axis was extracted. The root mean square error (RMSE) is used to compare the difference between the computed centroid and the ground truth. These groups of images contain both straight and curved roads. To this end, the RMSE for the sampling data was 1.275 pixels. This statistic indicates that the proposed method has an accurate response in estimating the centroid. Figure 10 shows the results for both centroids, and the ground truth in blue and the estimated shown in red. Since the detection of the centroid depends fully on the lane marking detection, the 1.275 RMSE value indicates the feasibility of the proposed lane marking detection strategy for intelligent vehicle applications.

#### 4.2.2. Road Geometry Parameters

**Figure 11:**
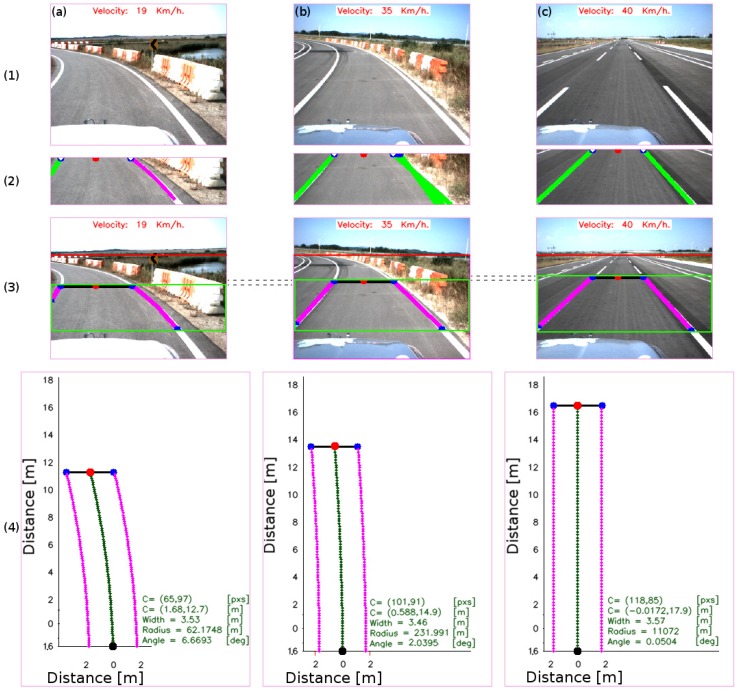
Lane region and parameters estimation. (**a**–**c**) show the estimation result of the road geometry model for a set of frames, at different speeds of the vehicle and their respective result at each step (rows 1–4). The first row shows the set of input images taken at different speeds. The second row shows the lane marking detection results (lines in **green** or curves in **magenta**), the extracted centroid of the lane marking (circled in **red**), and the ending point of the detected lane marking (circled in **blue**). The small circle in **white** indicates the line fitting model extracted to define the lane region. The third row shows the road geometry estimation within the image. The line in **black** represents the lane width (in pixels), the line/curve in **magenta** shows the lane marking which defines the region located in front of the vehicle. It can be noticed that the lane region increases as the speed of the vehicle increases, see the gaps between images (the dashed line in **black** between images). The fourth row shows the lane region detection in the real world using the model shown in Figure 2 and Figure 7. The point in **red** shows the estimated centroid. Points in **blue** show the ending point for each detected lane marking. The dashed **green** line shows the estimated current vehicle trajectory along the centerline of the lane between the rear-axle position and the estimated centroid of the lane (vehicle heading angle). The line in **black** shows the width of the lane, computed as the difference between the ending points. The region within the set of lines in magenta describes the lane region within the collision risk region estimate at each frame.

Next, the deflection angle, the chord length, the vehicle heading angle, and the lane width are evaluated. Figure 7 shows the simple circular curve road model used to extract the road parameters. The deflection angle (*θ*) is the angle formed along the chord length ($d_{a}$). The chord length is the distance computed between the estimated centroid (circled in red in Figure 7 and Figure 11) and the vehicle rear-axle position (circled in black in Figure 7 and Figure 11). The vehicle heading angle (*δ*) is the angle formed between the vehicle centerline and the road tangent line. Row 1 in Figure 11 shows a set of frames along straight and curved roads, row 2 shows the result of the lane marking detection, row 3 shows the iteratively road geometry model estimation within the input frame, and row 4 shows the lane region detection in the 3D environment at the ground level.

In that sense, the research result in [7] was used to evaluate the proposed method. This was done as the data for the tested road were collected using both laser and camera sensors simultaneously. As mentioned previously, the data were gathered at the Korea Automobile Testing & Research Institute (KATRI); consequently, extraction of the ground truth for the full path was not possible. This evaluation method is used due to the accuracy of laser sensors. Research in [21,22,23] introduced the use of laser scanning to solve the problem of visual localization within LiDAR maps, extraction of road markings and lane detection respectively. To this end, Table 3 shows the computed RMSE value between the laser and camera.

**Figure 12:**
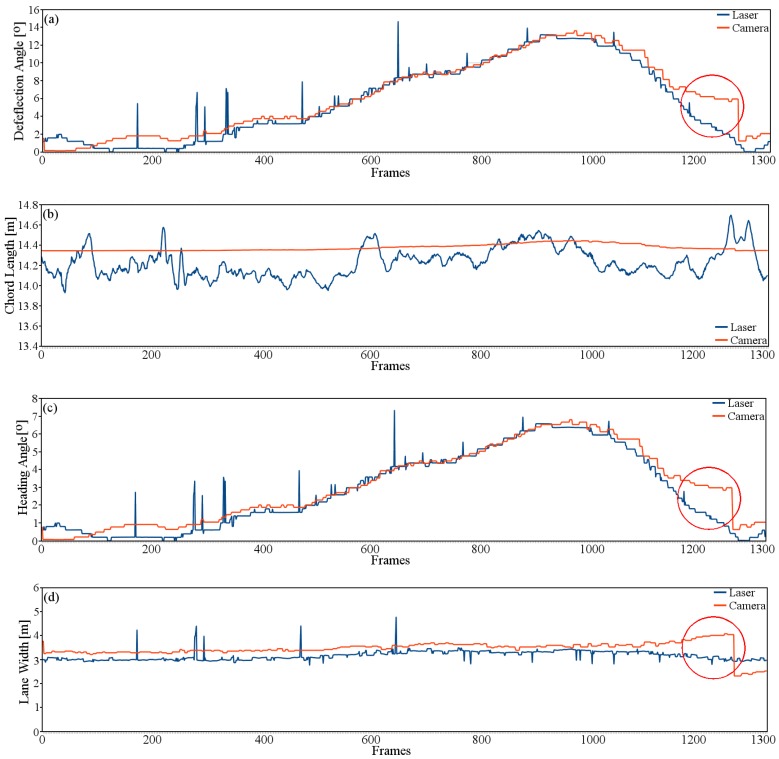
Road parameters evaluation using laser and camera, laser data in blue color while camera data is in orange color. (**a**) shows the deflection angle comparison; (**b**) illustrates the chord length comparison; (**c**) shows the vehicle heading angle; and (**d**) shows the lane width comparison. Circles in **red** shows the computed error due to abrupt change in illumination.

Figure 12 shows the deflection angle (*θ*), chord length (d${}_{a}$), the vehicle heading angle (*δ*) and the lane width estimated for both sensors. Laser and camera results are shown in blue and orange colors, respectively.

The circles in red show the response of the algorithm when changes in the illumination due to sunlight are presented. Instead of developing an algorithm to solve this problem due to strong sunlight (which increases the computing time), a merging strategy based on laser-vision will be proposed. However, further strategies will be implemented in order to extract the ground truth by adding a position sensor within the vehicle steering column.

### 4.3. Comparative Analysis

The proposed method is compared with the latest research. It should be noted that the information in the tables is related to the best performance results in each study. Additionally, the researchers proposed different strategies to solve the problem, making it difficult to compare results.

Table 4 shows the research results given in [4,5,13,24]. All of these approaches show good performance—over a 90% detection rate. However, it should be emphasized that the number of frames used to test their algorithm could affect the results. To test the proposed algorithm, 3781 frames were used, while Mammeri et al. used 912 frames and Du et al. used 350 for the quantitative comparison from a total of 2000 frames. Jung et al. mentioned that they recorded road video for the total time of 1 h and 40 min; however, they do not indicate the number of frames used in their best performance. Guo et al. used a set of 2132 frames in their best performance. On the other hand, the road environment, weather condition, and night or day conditions also affect the measurement. To test all of these algorithms, urban roads were used. However, it should be noted that the dataset used to test the proposed method contains barriers, bushes, shadows, spilled markers, and sand the along the lane marking, as well as different types of markers (see Figure 9), which increases the degree of difficulty at the moment of detection. To this end, authors in [4,5,13] show a better performance than the proposed method. In the case of [24], our proposed method shows better performance.

Comparing these algorithms in the presence of different marker types (channeling lines, stop lines, crosswalk), the presented strategy shows a robust response (see Figure 9). Mammeri et al. indicated that their algorithm in cross-roads will encounter some problems in the detection of lane markings. Authors refer to the case where crosswalk markings were recognized as lane markings (Figure 12c in [4]). Du et al. mentioned that their algorithm does not work properly in some regions (see Figure 27b, zebra crossing or crosswalk, in [13]). Jung et al. and Guo et al. did not show results related to the mentioned marker type regions. Mostly, the given errors are related to the edge extraction step. In other words, the edges are used as a feature without taking into consideration the lane marking width, reflecting the use of the edge (from crosswalks or traffic signs), or road markers (such as arrows or text) as a feature. On the contrary, the presented idea takes the size of the lane marking into consideration, reducing the error given by markers of a different type. The strategy presented in this approach shows a better performance in terms of computing time, which is crucial for real-time applications. The data acquisition interval for each algorithm at an assumed vehicle speed of 100 km/h is shown in the fifth column of Table 4. It can be seen that the proposed algorithm can gather data approximately every 0.8 m between frames.

The computing time improvement in this approach is related to the factors described below. The method efficiently extracts the bounded region of interest within the frame. The ROI in this method is dynamic and vehicle speed-dependent in a range of (256,(68–105)) pixels in size for speeds in a range of [0–100] km/h. In contrast, the latest research using different strategies is relatively time-consuming. For example, the authors in [4] resized the image to 640 × 240 prior to the implementation of their approach. Researchers in [13] used the bottommost part of the input image in other words, an image size of 640 × 240. Authors in [24] used an image of dimensions 856 × 240, while researchers in [5] used a spatiotemporal image strategy in which processing time should be affected by the camera frame rate. On the other hand, the RANSAC algorithm is applied if and only if the line fitting is insufficient to definitively model the lane marking within the already defined cluster (see Figure 5, sixth row). This is because it was statistically observed that a robust linear fit can be found 95% of time. Consequently, the time-consuming RANSAC algorithm is only used in rare events when it is absolutely necessary for discrimination. However, it should be noted that for the case of tight left and right turns, RANSAC plays an important role. For example, in [13], authors used simultaneous line/hyperbola road models, which can degrade the algorithm response time. Guo et al. used the RANSAC algorithm along with the least square method. Although the authors detect left and right markings independently, the number of points within the pool and the number of iterations will affect the lane marking detection results. Finally, instead of repetitively performing color probability computation during runtime to compute the metric according to the RGB and S channels, a look-up table was created for each color channel according to their respective PDF. The processing time information is shown in Table 5 and Table 6.

**Figure 13:**
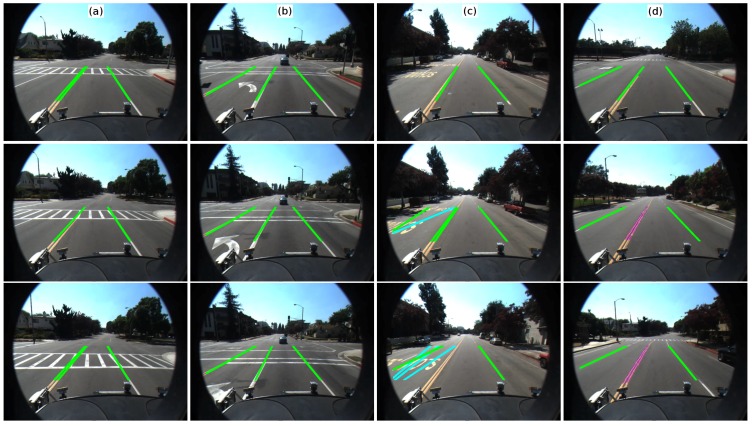
Lane marking evaluation using Caltech dataset. (**a**) shows the result of the proposed method in the presence of crosswalk areas; (**b**) shows the result for the case of stop lines and arrow; (**c**) shows the result of the wrong detection due to the text on the road; and (**d**) shows the error in detection given by double lane marking. It happens because of the clustering label both on the same cluster, due to the gap between the lane marking.

In order to verify the reliability of the proposed lane marking detection, the Caltech Lanes Dataset [9] was used to test the algorithm. The results are shown in Figure 13. It should be noted the difference in both sensor setup as well as field of view of the camera. In the proposed method, the lane ahead of the vehicle is mostly covered within the image, while the Caltech data set covers a larger area. Although the settings in the algorithm were not adjusted, the results showed a good response in the presence of crosswalk areas, arrows, as well as stop lines (Figure 13a,b, respectively). With respect to the traffic signs on the road, the algorithm failed in regions where the width of the signs was within the distance threshold used in our method to differentiate lane marking (see lines in magenta in Figure 13c). On the other hand, it was mostly double lane markings were not correctly detected due to the clustering process. This problem happened because the distance between two lane markings was not enough for the method to correctly separate them. As a consequence, both lane markings were labeled as the same group (see circles in magenta along the double yellow lane marking in Figure 13d). Then, the next step (line/curve fitting model) rejected the cluster as a lane marking since the sum of the error for both line and curve models was greater than the proposed threshold. Similarly, broken lane markings in some cases were not detected due to the number of extracted midpoints. In the proposed method, due to the sensor setup and the field of view, the algorithm considered a lane marking candidate if at least five midpoints were extracted. In the Caltech dataset, in some cases, three midpoints define a broken lane marking. Similarly, lane markings located at both sides of the laser (near the curbs) were not detected because PDF training data does not consider the pixels belonging to lane marking. To this end, the performance of the proposed algorithm using the Caltech data set is described as follows: specificity of 0.64, sensitivity of 0.95, accuracy 0.79, false positive per second rate of 0.93, and false negative per second rate of 4.64 at processing time of 12.13 ms. The program was built in C++ and compiled in Ubuntu 16.04 using an Intel Core i7-4770MQ, 2.40 GHz, with 8 GB of RAM, GeForce GT 750M/PCIe/SSE2. In the current implementation, the color pixel detection is implemented in CUDA (Compute Unified Device Architecture). The encountered problems will be solved in the current ongoing laser-camera hybrid method.

Finally, it should be noted that the current algorithm was not tested under environmental conditions such as rainfall, foggy weather or nighttime. In order to overcome this problem, additional improvement will be done by analyzing the reflectance of pavement-marking under these conditions. In the case of camera sensors, the idea will be focused on the approach of enhancing visibility [25,26]. In the case of laser sensors, the analysis will be based on the surface patterns characteristic of the road in the presence of rain water [27] or fog [28]. Research in [7] showed that, in the case of nighttime, the laser demonstrates good performance.

## 5. Conclusions

In this paper, a vision based real-time lane marking, lane understanding application was presented for use in intelligent vehicles. The aim of this paper has been three-fold:

- To introduce an automated method for extracting the region of interest based on the relationship between the vehicle speed and the typical stopping distance within the image. As a result, the image is sectioned into three regions: information above the horizon, a zero collision risk, and a collision risk region.
- To propose a real-time lane marking detection strategy combining edge and color features, which uses the probability that the extracted features define a lane marking, as well as a hierarchical fitting model. As a result, the method is able to detect lane markings with an accuracy of 96.84% at an average processing time of 28.30 ms, for a speed range of [5–45] km/h. Similarly, the algorithm solves the problem given by traffic marking signal types such as channelizing lines, stop lines, crosswalks, and arrows.
- To estimate the lane region and the vehicle heading angle by using the road geometry model and the lane centroid information.

Although a classification method based on a probability density function was used in order to improve the response in the presence of strong shadows or changes in illumination (which takes into consideration yellow, white and lane marking with shadows), there will be future improvements. These improvements will be made by developing a laser-camera hybrid method; the results of the ongoing process are shown in [29]. Similarly, the proposed method will be improved by taking different environmental conditions into consideration.

## Figures and Tables

**Table 1:** Lane marking detection rate for a solid lane marking type.

Types ^a^	Frames	TP	FP	FN	TN	St [%]	Sp [%]	A [%]	FPs	FNs
Total	3781	5891	148	234	5851	96.17	97.53	96.84	0.92	1.46
Single	3781	3781	148	138	7961	96.45	98.17	97.22	0.92	0.86
Double	1055	2014	0	96	2683	95.45	100	97.99	0	2.15

${}^{a}$ Note: TP stands for true positive, FP stands for false positive, FN stands for false negative, TN stands for true negative, St stands for sensitivity, Sp stands for specificity, A stands for accuracy, FPs stand for false positive per second, and FNs stand for false negative per second.

**Table 2:** Line/curve fitting error in pixels.

	Average	Standard Deviation	Maximum Value
Line	4.34	3.25	14.88
Curve	3.81	0.336	4.39

**Table 3:** Laser-camera root-mean-square error (RMSE) evaluation.

	Deflection Angle [${}^{\circ }$]	Chord Length [m]	Vehicle Heading Angle [${}^{\circ }$]	Lane Width [m]
RMSE	1.28	0.20	0.63	0.40

**Table 4:** Comparison of different techniques in drivable region detection.

Authors ^a^	Frame No.	DR [%]	FDR [%]	PT [ms]	DA [m]
Proposed method	3781	96.17	2.47	28.30	0.77
Mammeri et al. [4]	912	100.00	3.60	≫32.31 **	≫0.90
Du et al. [13]	350	98.50	2.20	120.04	3.33
Jung et al. [5]	N/D	98.31	N/D	116.82	3.24
Guo et al. [24]	2132	94.79	7.77	40.00	1.10

${}^{a}$ Note: DR stands for detection rate, FDR stands for false detection rate, PT stands for processing time, N/D stands for no data, and DA stands for data acquisition at assumed speed of 100 km/h. ** Authors do not discuss information regarding the processing time. Consequently as the information relates to the edge segmentation results (Table 2 in [4]).

**Table 5:** Computational time for each task in milliseconds.

Distance	ROI ^a^	Color	Edge	Mid-Points	Clustering	Fitting	Heading	Total
0.0407	0.0807	5.8629	6.2400	0.7989	11.788	2.4640	1.0269	28.3021

${}^{a}$ Note: ROI stands for region of interest extraction.

**Table 6:** Computing time in milliseconds.

Total	Standard Deviation	Max. Time
28.3021	7.003	48.99
